# Clustering analysis of tumor metabolic networks

**DOI:** 10.1186/s12859-020-03564-9

**Published:** 2020-08-25

**Authors:** Ichcha Manipur, Ilaria Granata, Lucia Maddalena, Mario R. Guarracino

**Affiliations:** 1 National Research Council, Institute for High-Performance Computing and Networking, Via P. Castellino 111, Naples, 80131 Italy; 2 HSE - National Research University Higher School of Economics, LATNA Laboratory, 13 Rodionova Ulitsa, Nizhny Novgorod, Russia

**Keywords:** Metabolic networks, Network simplification, Networks clustering

## Abstract

**Background:**

Biological networks are representative of the diverse molecular interactions that occur within cells. Some of the commonly studied biological networks are modeled through protein-protein interactions, gene regulatory, and metabolic pathways. Among these, metabolic networks are probably the most studied, as they directly influence all physiological processes. Exploration of biochemical pathways using multigraph representation is important in understanding complex regulatory mechanisms. Feature extraction and clustering of these networks enable grouping of samples obtained from different biological specimens. Clustering techniques separate networks depending on their mutual similarity.

**Results:**

We present a clustering analysis on tissue-specific metabolic networks for single samples from three primary tumor sites: breast, lung, and kidney cancer. The metabolic networks were obtained by integrating genome scale metabolic models with gene expression data. We performed network simplification to reduce the computational time needed for the computation of network distances. We empirically proved that networks clustering can characterize groups of patients in multiple conditions.

**Conclusions:**

We provide a computational methodology to explore and characterize the metabolic landscape of tumors, thus providing a general methodology to integrate analytic metabolic models with gene expression data. This method represents a first attempt in clustering large scale metabolic networks. Moreover, this approach gives the possibility to get valuable information on what are the effects of different conditions on the overall metabolism.

## Background

Biological data produced by high throughput experiments, and in particular by Next Generation Sequencing technologies are being accumulated in publicly available databases. Multi-year research projects, such as The Cancer Genome Atlas (TCGA) [1], are producing petabytes of data. Besides this type of initiatives, there are many research projects focused on extracting knowledge from experiments, and they are storing resulting metadata in knowledge-based repositories. One of these projects is the Human Metabolic Atlas (HMA) [2], which has been accumulating genome-scale metabolic models for different healthy and cancer tissues. Such models describe in analytic format the knowledge about specific tissues metabolism. From the integration of such different sources, it is possible to obtain a knowledge-based characterization of patients with different cancer sub-types.

In the case of Genome Scale Metabolic (GSM) models, this integrative approach has been used in several studies [3–5]. It usually involves gene expression data and metabolic models, to compare two conditions (e.g., healthy vs diseased), highlighting an average behaviour of a group of patients with respect to another. In order to overcome this limit, we decided to model patients separately, obtaining a network model for each of them. Starting from this representation of the cohort, we proposed a supervised learning technique to produce predictive mathematical models to classify diseases and their sub-types [6].

Following these ideas, we decided to devise a clustering technique for network data. Again, each patient is represented in form of a network integrating gene expression data and a GSM model. We obtain a representation of the networks based on a probability distribution of their shortest paths, and an *ad-hoc* distance based on Shannon-Jensen divergence is used to compute their pairwise distances. Since the number of nodes in such networks is in the order of thousands, we explored a simplification technique [7, 8] that helps in reducing the computational time needed for the distance computation. Using the mutual distances among networks, it is natural to represent them in a similarity matrix, on which spectral clustering [9] can be used to characterize the classes of patients. There are two main advantages of this solution. First, modeling each sample in the dataset using data from both a metabolic model and a gene expression experiment, provides more information. Then, representing the data in the form of networks permits to obtain a quantitative model that retains information about the cross-talk existing among the different pathways and modules of the human metabolism.

**Fig. 1:**
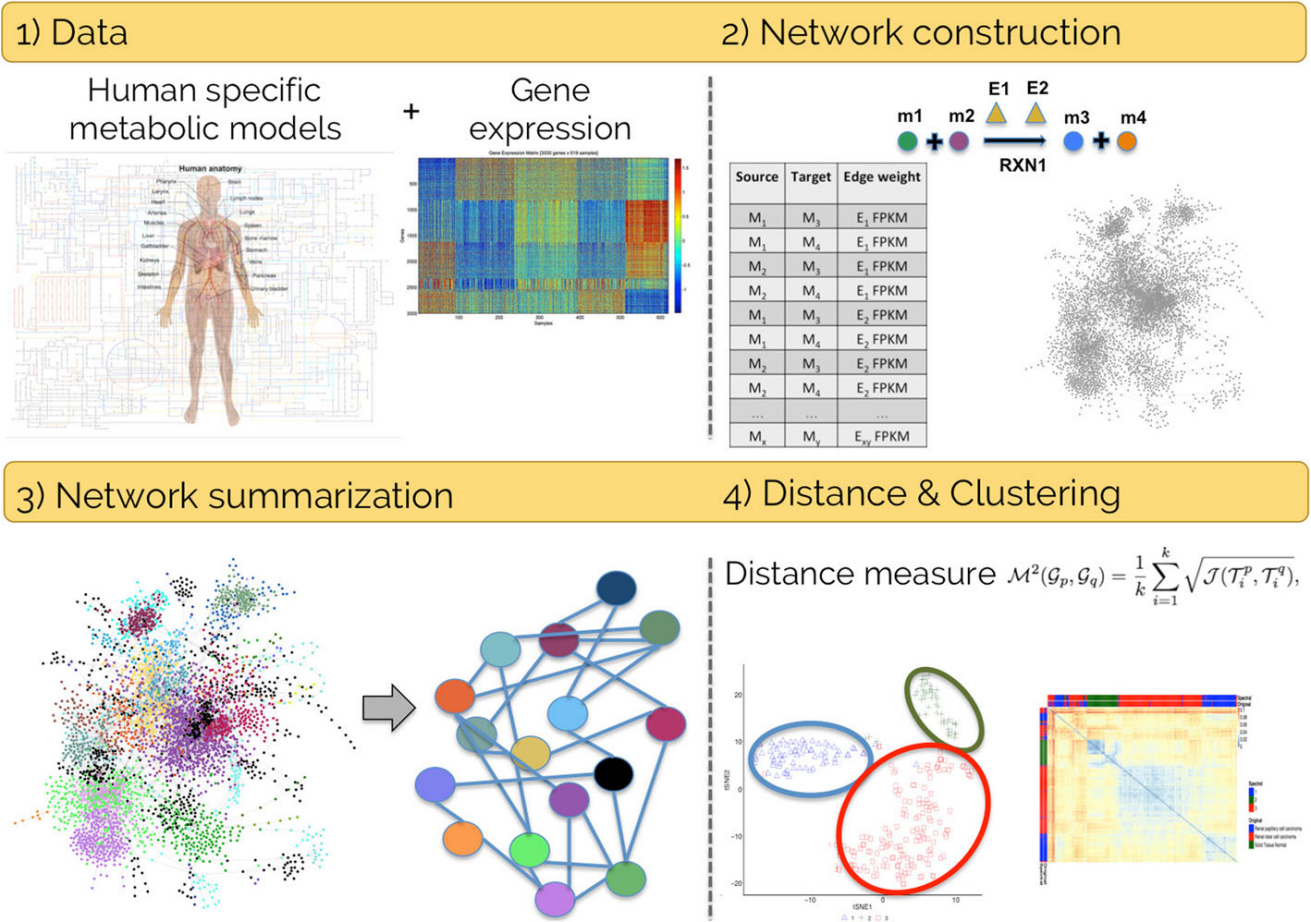
Workflow Schematic representation of the workflow used in the present study. 1) The input data are the tissue/cell metabolic models and the gene expression values. 2) The network is constructed connecting reagent and product metabolites involved in the same reaction, catalyzed by one or more enzyme, whose expression represents the weight of the edges. Multiple edges connecting two nodes are then merged into single edges. 3) The whole network is summarized through clustering and supernodes collapse. 4) The distance among summarized networks is calculated using the $\mathcal {M}^{2}$ metric, and used to cluster the samples

The overall work-flow used in the present study is schematically shown in Fig. 1. Its main four main steps are detailed in the “Materials and methods” section.

## Materials and methods

### Data

Gene expression data of breast cancer from microarray experiments are publicly available in the NCBI Gene Expression Omnibus database [10] (GSE78958). Raw CEL files were imported, corrected, transformed, and normalized using GEOquery [11] and Affy [12] R packages. Probe ids were mapped to relative gene symbols using the annotation file “hgu133a2.db”. RNA sequencing data of breast cancer (Project TCGA-BRCA), lung cancer (Projects TCGA-LUSC and TCGA-LUAD), and kidney cancer (Projects TCGA-KIRC and TCGA-KIRP) collected into the Genomic Data Commons Data Portal (https://portal.gdc.cancer.gov) were downloaded in the form of FPKM normalized read counts. As summarized in Table 1, the **Breast Microarray dataset** contains 418 samples of four intrinsic molecular subtypes based on BreastPRS [13, 14]: Basal-like (99 samples), HER2-enriched (50 samples), Luminal A (226 samples), and Luminal B (43 samples). The **Breast RNAseq dataset** contains 401 samples of two intrinsic molecular subtypes based on PAM50 [15]: Luminal A (200 samples), and Luminal B (201 samples). The **Lung dataset** contains 337 samples divided into three groups: Adenocarcinoma (159 samples), Squamous carcinoma (150 samples), and Solid tissue normal (28 samples). The **Kidney dataset** contains 299 samples divided in three groups: 159 samples of clear cell Renal Cell Carcinoma (ccRCC or KIRC), 90 samples of Papillary Renal Cell Carcinoma (PRCC or KIRP), and 50 samples of Solid tissue normal.

**Table 1:** Number of samples per class (#) for the four datasets

Breast Microarray		Breast RNAseq		Lung		Kidney	
Class	#	Class	#	Class	#	Class	#
Basal-like	99	Luminal A	200	Adenocarcinoma	159	ccRCC	159
HER2-enriched	50	Luminal B	201	Squamous carcinoma	150	PRCC	90
Luminal A	226			Solid tissue normal	28	Solid tissue normal	50
Luminal B	43						
TOTAL	418		401		337		299

For each type of cancer under study, the corresponding metabolic network was downloaded from the HMA database [2] in the compressed Systems Biology Markup Language (SBML) format [16]: breast cancer INIT model [17], lung tissue, and kidney tissue models [18]. The metabolic models were imported, and the relative stoichiometric matrices were extracted in the R environment using the sybilSBML package.

Raw expression data are considered as basic data for comparison. They are a subset of the whole gene expression data obtained by selecting the metabolic genes. We refer to these data with the term “Expression” in the experimental results.

### Network construction

**Fig. 2:**
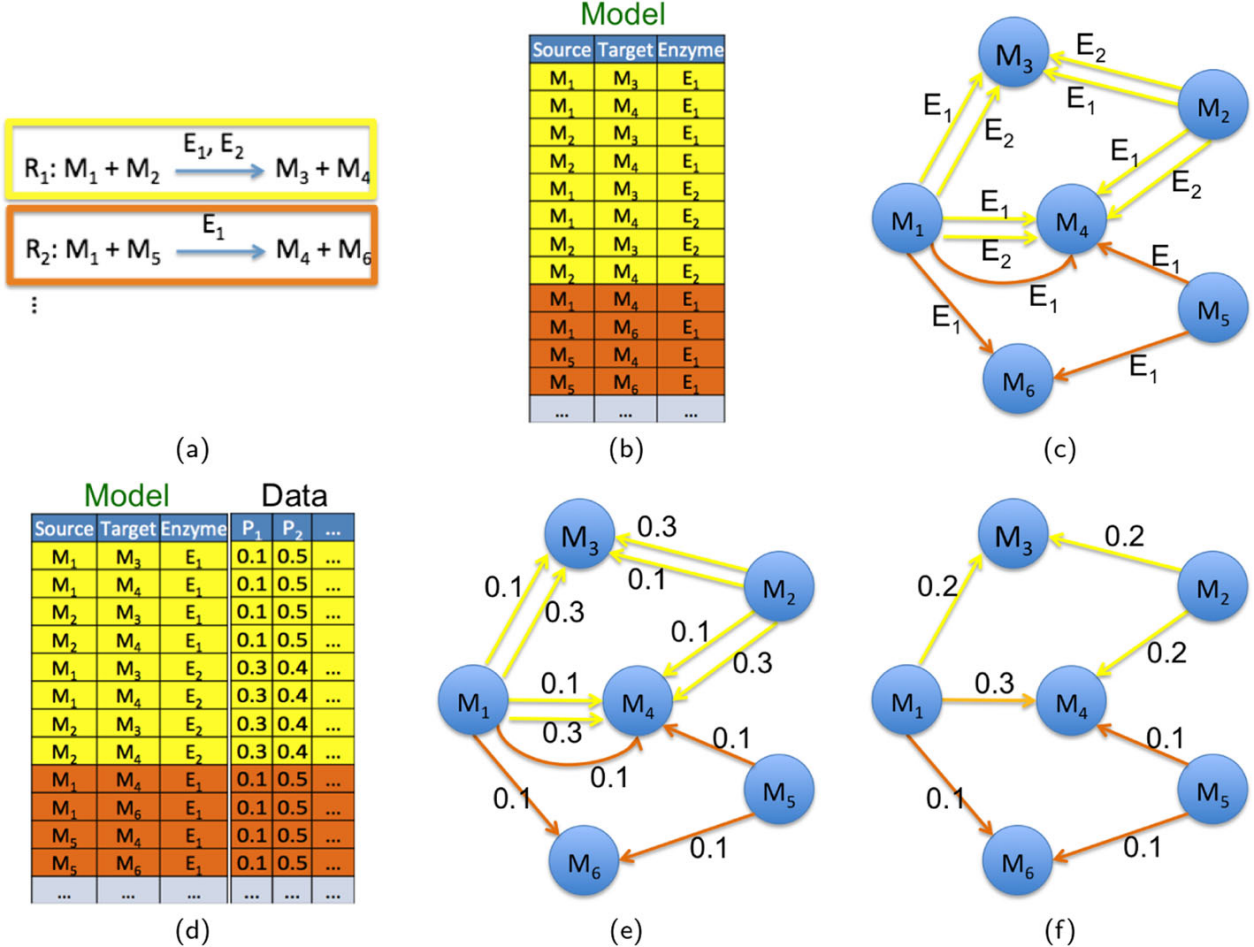
Network construction The graphs are generated considering the metabolites as nodes, with edges connecting metabolites involved in the same reaction, one as a reagent and the other one as the product. **a** Representation of a metabolic model as a set of reactions. **b** For each reaction and each gene/enzyme catalyzing it, we consider all the (source, target) couples of involved metabolites. **c** The network for the metabolic model is obtained by linking each node representing a source metabolite with the node representing the corresponding target metabolite, with as many links as there are enzymes catalyzing the reaction. **d** Data for each patient (e.g., patient P_1_) is given by the relative gene expression values of the sample, **e** that are used for weighting the corresponding edges. **f** The obtained multigraphs are reduced to simple graphs, where weights are computed by averaging the gene expression values in the same reaction and adding these means in the case of different reactions. For example, the edges between the source metabolite M_1_ and the target metabolite M_4_ are reduced to a single edge whose weight (0.3) is computed by adding the average (0.2) of the weights for the two yellow links with the weight (0.1) of the orange link

The graphs (throughout the paper, we will use the terms graph and network interchangeably) were generated considering the metabolites as nodes, with edges connecting metabolites involved in the same reaction, one as a reagent and the other one as the product, as illustrated in Fig. 2. Given the metabolic model (Fig. 2a), for each reaction and each gene/enzyme catalyzing it, we consider all the (source, target) couples of involved metabolites. (Figure 2b). The network for the metabolic model is obtained by linking each node representing a source metabolite with the node representing the corresponding target metabolite, with as many links as there are enzymes catalyzing the reaction (Fig. 2c. Data for each patient is given by the relative gene expression values of the sample Fig. 2d), that are used for weighting the corresponding edges (Fig. 2e). The obtained multigraphs (i.e., graphs where two nodes may be linked by more than one edge) are finally reduced to simple graphs (referred to as “Whole graph” in the results), where weights are computed by averaging the gene expression values in the same reaction and adding these means in the case of different reactions (Fig. 2f). In general, the mapping of enzymes to the reactions represents a difficult task since it is not a one-to-one association, as multiple enzymes can catalyze the same reaction as well as one enzyme can catalyze multiple reactions. We assumed that the weights of multiple enzymes catalyzing the same reaction can be simplified by averaging the expression values of their corresponding genes. Some of them work in complexes and some others are alternative catalysts, but we considered all their expressions equally important for the regulation of the connection between a substrate and a product. Instead, the relationship between genes catalyzing different reactions and linking the same metabolites can be considered as a Boolean OR, as the path from one metabolite to the other is alternatively defined by one reaction OR the other, and thus simplified by summing their values, as suggested in [19].

The same metabolite can have more than one cellular compartment localization fulfilling different functions; thus, we considered it as a different metabolite in each compartment Reactions not catalyzed by any enzyme were not considered and disconnected nodes were excluded as well. Indeed, networks representing different patients differ only for the weights of their links, and this information would be absent in both the above cases.

The recurrent metabolites (such as H2O and CO2; see Additional file 1 for a complete list) were excluded from the network. Indeed, they are functional groups, cofactors, and carriers for electrons transferring, and cannot be intrinsically considered compounds; therefore, their connections would give rise to an unrealistic definition of the paths and their lengths, as suggested in [20]. The metabolite-based networks were generated through an in-house R script, giving rise to 3254, 3380, 3959, and 4022 nodes for the Breast Microarray, Breast RNAseq, Lung, and Kidney networks, respectively. The networks are then partially processed using igraph R package [21]. Further details concerning the data networks are reported in Table 2.

**Table 2:** Network details for the four datasets

	Breast Microarray	Breast RNAseq	Lung	Kidney
#Genes	1931	2622	2612	2801
#Nodes	3254	3380	3959	4022
#Nodes in largest connected component	2848	3041	3537	3623
#Edges	21902	29536	41038	43622

### Network summarization

Given a network $\mathcal {G} = (V,E,W)$, where *V* and *E* are the set of all nodes and edges in $\mathcal {G}$, respectively, and *W* is the weight matrix, its summarization into *supernodes* was obtained as described in [7]. Briefly, given a partition of the *l* nodes of the network $\mathcal {G}$ into *k* clusters, a matrix *Q*=[*q*_1_,*q*_2_,...,*q*_*k*_]∈*R*^*l*×*k*^ is considered, consisting of a set of indicator vectors *q*_*j*_ that represent the membership relationship for cluster *j*,*j*=1,…,*k* (i.e., *q*_*j*_=(*q*_1,*j*_,…,*q*_*l*,*j*_)^*T*^, where, for all *i*, *q*_*i*,*j*_=1 if node *i* is in cluster *j* and *q*_*i*,*j*_=0 otherwise). The summarized network $\mathcal {G}_{s} = {\left (V_{s},E_{s},W_{s}\right)}$ is formed by merging the nodes in each of the *k* clusters into a *supernode*; the weight matrix *W*_*s*_ is obtained by *W*_*s*_=*Q*^*T*^*WQ*. Further details are given by the authors in [7].

In our approach, network summarization is applied to the largest connected component of each network (see Table 2), having experienced that the other disconnected components contained too few nodes, less than 1% of the largest connected component (see Additional file 2). For computing the initial partition of each network into *k* clusters, we adopted spectral clustering; a well know algorithm used for cluster analysis [9]. It uses a representation of the dataset in terms of mutual distances among the samples, and therefore is well suited for networks.

Spectral clustering uses as a main tool the *Laplacian matrix*$\mathcal {L}=\mathcal {D}-A$ of a graph $\mathcal {G}$, where *A* is the adjacency matrix of $\mathcal {G}$ and $\mathcal {D}$ is a diagonal matrix, where $\mathcal {D}_{i,i}$ is the degree of node *i*,*i*=1,…,*l*. The eigenvectors *x* and eigenvalues *λ* of $\mathcal {L}$ are obtained by solving $\mathcal {L}x = {\lambda }x$. Finally, the eigenvectors of the graph Laplacian $\mathcal {L}$ that correspond to the *k* smallest eigenvalues are used as features for a clustering algorithm, where each row of that matrix is a representation of the corresponding sample in the dataset.

In our implementation, we used the spectral.clustering function of the fcd (Fused community detection) package in R [22]. For each dataset, clustering was performed on the unweighted adjacency matrix of its graphs, that is common to all of them (all the networks within a dataset have the same nodes and edges; only the edge weights change, based on gene expression values that are different for each sample).

### Distance and clustering

The final step of our approach involves the calculation of distances between the summarized networks, in order to cluster the patient samples. In [23], we represented networks using a set of metrics obtained by computing distances between probability distributions of network topological properties, which were an extension of the definitions introduced in [24–26]. In this study, we use the distances calculated between *transition matrices*$\mathcal {T}^{r}$ of different networks $\mathcal {G}_{r}$, whose element $\mathcal {T}^{r}_{i}(j)$ is the probability of node *i* to be reached in one step by a random walker located in node *j*. Therefore, $\mathcal {T}^{r}$ is the adjacency matrix of $\mathcal {G}_{r}$ rescaled by the degree of each node and contains local information about the connectivity of $\mathcal {G}_{r}$.

Given two networks $\mathcal {G}_{p}$ and $\mathcal {G}_{q}$, let their transition matrices be $\mathcal {T}^{p}$ and $\mathcal {T}^{q}$, respectively. Averaging over all *k* supernodes of the summarized networks, we defined the network distance $\mathcal {M}^{2}$:

1 $$ \mathcal{M}^{2}\left(\mathcal{G}_{p},\mathcal{G}_{q}\right) = \frac{1}{k}\sum_{i=1}^{k} d_{JS}\left(\mathcal{T}_{i}^{p},\mathcal{T}_{i}^{q}\right) = \frac{1}{k}\sum_{i=1}^{k} \sqrt{\mathcal{J}\left(\mathcal{T}_{i}^{p},\mathcal{T}_{i}^{q}\right)},   $$

where *d*_*JS*_ is a metric known as *Jensen-Shannon* distance [27], defined as the square root of the Jensen-Shannon divergence $\mathcal {J}$ of the two distributions [28]. Using the distance in Eq. (1), each network in the dataset can be represented by the vector containing the distances from all other elements.

The square matrix containing in each row the vector representing a sample from the dataset is usually called the *Gram matrix* or *distance matrix*.

For obtaining the final clustering results of the proposed approach, we applied spectral clustering from the sklearn package in Python [29] to the distance matrices computed for the summarized networks.

## Results and discussion

### Analysis of summarized networks

The summarization of the whole network through spectral clustering and supernodes collapse gave rise to different sized networks, depending on the number *k* of clusters that were set (Fig. 1 step 3). Inspired by [8], we chose six values of *k*, ranging between 50 and 300, which is approximately less than 10% of the nodes of the whole graphs, and evaluated the clustering performance on the distance matrices (see Additional file 3). A range tighter than the one in [8] was set to better analyze small differences. The best performance was obtained using 300 supernodes for all the datasets except the kidney, which shows the best results with 250 supernodes.

**Fig. 3:**
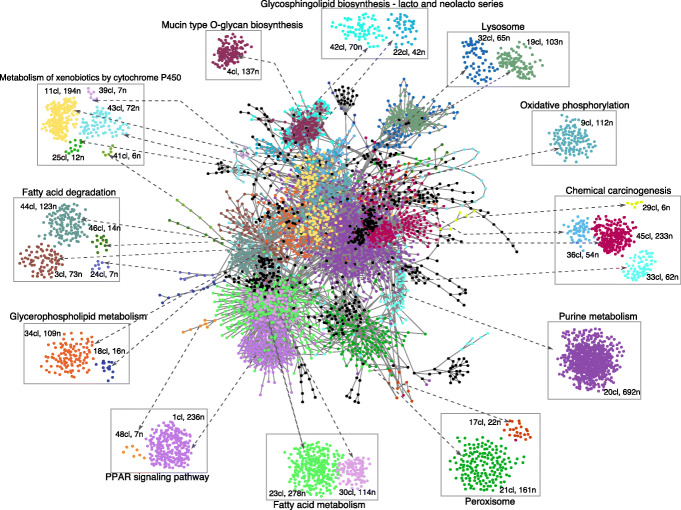
The kidney metabolic network (center), represented in Cytoscape v3.7.1 shows 50 clusters detected by spectral clustering. The KEGG terms for the enzymes belonging to the clustered nodes were enriched using the clusterProfiler package [41] in R. Insets in the periphery show details of 28 clusters derived from the network, where n is the number of nodes and cl is the cluster number. The first KEGG term significantly enriched by individual clusters is shown above each inset

As an example of possible evaluation of the clusters from a biological point of view, in Fig. 3, we show the KEGG pathways enriched by the enzymes from the edges of all pairs of interacting nodes (metabolites) present in each of the 50 clusters of the kidney metabolic network (see Additional File 4 for details). The figure shows that the clustering of the whole network gives rise to communities of nodes and relative edges which have a defined biological function. The enriched terms containing the highest number of enzymes enriching a pathway are shown for the top 28 clusters, according to nodes number. Most of the terms are enriched by a single cluster or few of them, suggesting that almost each of the obtained clusters has a specific biological meaning. Some of the bigger clusters enriched terms as “Metabolism of xenobiotics by cytochrome P450” and “Chemical carcinogenesis”, which are well known to be involved in tumor metabolism and particularly in renal injuries. The kidney is the organ responsible for the elimination of drugs from the body, but it is also involved in drug metabolism through activity of cytochrome P450 (CYP) enzyme group and cross-talk with the liver [30]. Furthermore, due to its functions of filtering and reabsorption, the exposure to carcinogenic substances is much higher than other organs [31]. The most abundant cluster (692 nodes) enriches “Purine metabolism” pathway. Purines are involved in many biological processes, including immune responses and host–tumor interaction, and their metabolism changes continuously in response to cell demands; thus, it is a consequence that the alteration of the enzymes involved in this pathway, organized in dynamic multienzyme complexes called “purinosome”, occurs in severe diseases. In particular, purine metabolism involvement and nucleotide imbalance in tumorigenic processes has been largely demonstrated [32–34].

### Performance results

Several metrics exist for clustering. In the experiments, we consider an extended set of metrics often adopted for clustering evaluation [29, 35–38], to allow easier comparison with existing and newly proposed methods. They are described in detail in the Additional file 5 and summarized in Table 3, where we report their name (column Name), abbreviation (column Acronym), definition (column Computed as), possible values (column Codomain), and whether they should be minimized (*↓*) or maximized (*↑*) to have more accurate results (column Better if). Matlab scripts used for clustering evaluation are provided as Additional file 6.

**Table 3:** Metrics adopted for clustering evaluation

Name	Acronym	Computed as	Codomain	Better if
Rand’s Index	*RI*	$\frac {TP+TN}{TP+FP+FN+TN}$	[0,1]	*↑*
Adjusted Rand’s Index	*ARI*	$\frac {RI-E[RI]}{\max (RI)-E[RI]}$	[-1,1]	*↑*
Misclassification Rate	*MR*	$\frac {FP+FN}{TP+FP+FN+TN}$	[0,1]	*↓*
F-Measure	*F*_1_	$\frac {2 Precision \cdot Recall}{Precision + Recall}$	[0,1]	*↑*
Fowlkes-Mallows Index	*FMI*	$\frac {TP}{\sqrt {(TP + FP)\cdot (TP+ FN)}}$	[0,1]	*↑*
Cluster Accuracy	*CA*	$\frac {1}{n} \sum _{i=1}^{c} \max ({CP}_{i}|{GT}_{i})$	(0,1]	*↑*
Normalized Mutual Information	*NMI*	$\frac {MI}{\sqrt {H(CP) H(GT)}}$	[0,1]	*↑*
Adjusted Mutual Information	*AMI*	$\frac {MI-E[MI]}{\sqrt {H(CP) H(GT)}-E[MI]}$	[-1,1]	*↑*

(For details on the adopted metrics, see Additional file 5)

Performance results for the four datasets are reported in Table 4. Here, “Expression” refers to results achieved by spectral clustering applied to the gene expression data obtained in the first step of the proposed approach, “Whole graph” refers to results obtained by spectral clustering on the distance matrices of the networks constructed in the second step, and “Summarized graph” refers to the results achieved by the proposed simplified approach.

**Table 4:** Performance of spectral clustering algorithm on the four datasets

Clustering data	*RI*	*ARI*	*MR*	*F*_1_	*FMI*	*CA*	*NMI*	*AMI*
Breast microarray								
Expression	47.28	-2.06	52.72	43.70	44.66	44.02	13.84	10.84
Whole graph	67.30	26.25	32.70	49.77	50.28	55.98	30.10	27.56
summarized graph	66.05	26.09	33.95	52.40	52.45	53.11	19.96	19.00
Breast RNAseq								
Expression	53.05	6.21	46.95	60.99	61.90	62.59	10.08	8.14
Whole graph	52.81	5.62	47.19	52.85	52.85	62.09	4.30	4.11
Summarized graph	54.90	9.81	45.10	54.82	54.82	65.84	7.37	7.20
Lung								
Expression	89.79	79.17	10.21	88.11	88.12	94.07	78.31	77.83
Whole graph	89.56	78.73	10.44	87.91	87.92	93.77	75.94	75.02
Summarized graph	87.56	74.64	12.44	85.56	85.57	92.58	72.72	72.00
Kidney								
Expression	88.67	76.42	11.33	85.88	85.88	91.97	70.89	70.66
Whole graph	87.91	74.94	12.09	85.11	85.12	91.64	69.50	68.80
Summarized graph	88.09	75.42	11.91	85.52	85.56	91.64	70.00	68.80

(All values have been multiplied by 100)

For the Breast Microarray dataset, we observe that clustering gene expression data leads to poor overall performance. Indeed, RI values below 50% reveal very low accuracy and negative ARI values reveal that accuracy is even lower than the one that could be obtained by a random partitioning. At the same time, more than 50% of the times the wrong decision is taken (MR=52.72%). Similarly, all the other metrics confirm poor performance. This can be ascribed to the microarray technology used to quantify the gene expression, which is known to be less sensible for slight differences in expression measurements compared to the RNA-seq method used for the other datasets. Moreover, Bartlet et al. [39] investigated the classification of breast cancer into intrinsic molecular subtypes, showing that the classifications obtained using different tests were discordant in 40.7% of the studied cases. This could also justify the poor results obtained for clustering the raw expression data from the Breast RNAseq dataset. On the other side, we observe fairly improved performance for the Breast Microarray dataset when using both whole and summarized graph data. This may mean that considering metabolic interactions, rather than raw data, can help in capturing the differences between breast cancer subtypes.

This also holds true for the case of the Breast RNAseq dataset, even though with a small improvement as compared to the case of gene expression data.

For the Lung and Kidney datasets, clustering of raw data leads to quite high performance, as witnessed by low values of MR and high values for all the other performance metrics. Comparable performance is achieved using whole graphs, slightly better than using summarized graphs.

For all the datasets, the execution times for computing the distance matrices for summarized graphs strongly decrease (≈ 35 times less of the execution times for whole graphs).

Therefore, we can conclude that the proposed simplification proves to be beneficial for clustering “difficult” high-throughput data (e.g., those coming from imprecise technologies or having rather uncertain ground truth classification) and only minimally detrimental in the other cases. In all cases, it allows a strong reduction in execution times, making it feasible for big data analyses.

### Visual exploratory analyses

T-distributed Stochastic Neighbor Embedding (t-SNE) is a nonlinear dimensionality reduction technique that allows embedding of high-dimensional data for visualization in a low-dimensional space [40]. It models each high-dimensional sample by a two- or three-dimensional point in such a way that similar samples are modeled by nearby points and dissimilar samples are modeled by distant points with high probability. It is capable of retaining the local structure of the high-dimensional data, while also revealing some important global structure, such as the presence of clusters at several scales.

**Fig. 4:**
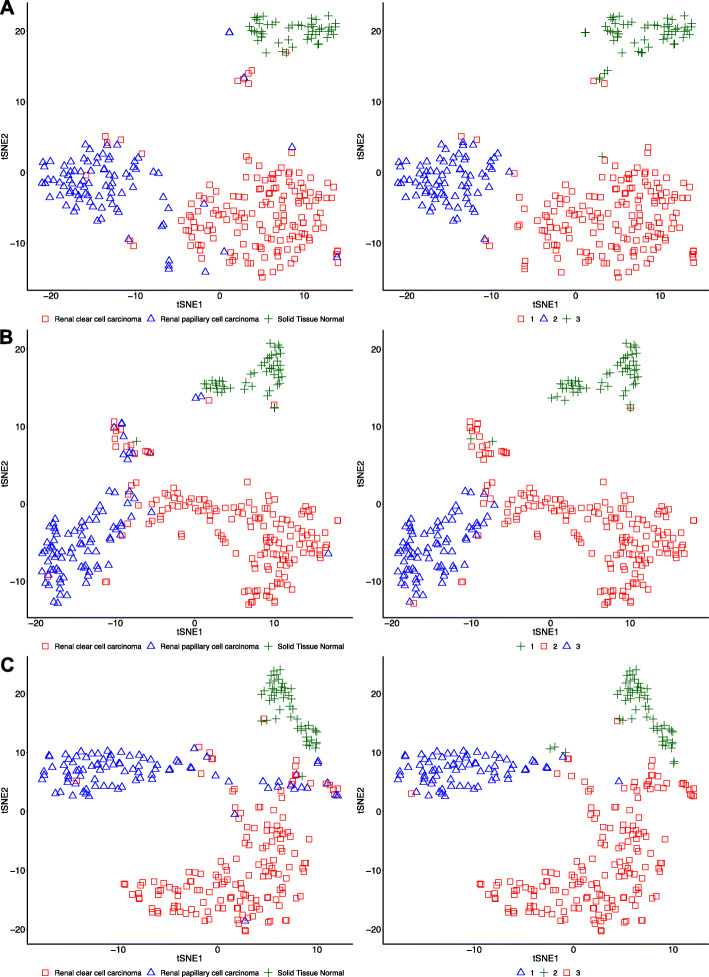
Kidney dataset t-SNE representation of gene expression data **a** and distance matrices obtained from whole **b** and summarized **c** graphs. The panel on the left shows the ground truth labels of the different classes and the right panel shows the labels assigned by spectral clustering

Figure 4 provides visual representations of the Kidney data mapped into the 2D Euclidean space by t-SNE. Data are colored to reflect the ground truth classification (left column) and the clustering results (right column).

The top row of Fig. 4 reports the visual representation for the gene expression data. Here, three clusters are obtained, basically consistent with the ground truth classification (left column). Only a few embedded points corresponding to ccRCC appear close to the PRCC cluster and *vice versa*. Moreover, a small of group of embedded points from the two carcinoma classes appears close to the solid tissue normal class. These few visual anomalies also reflect in the clustering results (right column), where the three clusters appear more uniform that the ground truth (i.e., no red point in the center of the blue cluster and no blue points in the center of the red cluster), consistently with the visual judgement.

The middle row provides the visual representation for the whole graph data. Here, the two carcinoma clusters are spatially contiguous, without marked separation (left column). The same can be said for the clustering results (right column), where most of the misclassified points appear spatially close to the cluster they are deemed to belong to (i.e., no blue point in the rightmost border of the red cluster and no blue points in the lower border of the green cluster). This shows that spectral clustering on these data fails like we would fail in judging based on the t-SNE visual representation.

The bottom row reports the visual representation for the summarized graphs. Similarly to the case of gene expression data, three spatially separated clusters can be identified, corresponding to the three different classes (left column). Only few carcinoma samples lye in an ambiguous region of the plane, at the intersection of the three clusters. Clustering assigns almost all of these few samples to the ccRCC class (right column), predicting the ambiguous plane region as belonging to this class.

The above analysis of visual representations obtained using t-SNE suggests that the proposed simplification leads to clusters that are better separated than in the whole graph case, and similarly to the gene expression case, thus confirming the comparable performance achieved. Analogous analyses carried out for all the considered datasets (see Additional file 7) confirm the achieved performance results.

**Fig. 5:**
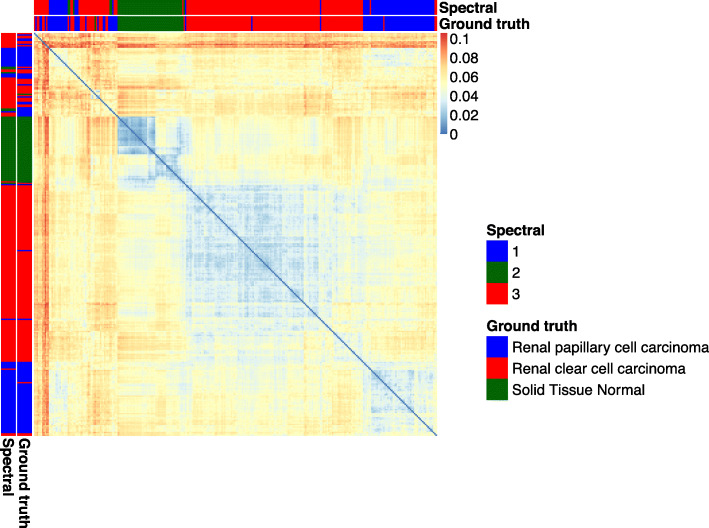
Heatmap representation of the clustering performed on the distance matrix of the Kidney summarized network. The patients are ordered according to the distance values. The labels from ground truth classification and from spectral clustering are shown. In both cases, three clusters are clearly visible, representing the three classes: PRCC (blue), ccRCC (red), solid tissue normal (green)

The heatmap in Fig. 5 is a color-based representation of the distance matrix obtained by the calculation of the $\mathcal {M}^{2}$ metric between the summarized graphs created for the Kidney project patients. The patients are ordered according to distance values. The labels from ground truth classification and from spectral clustering are shown. In both cases, three clusters are clearly visible, representing the three classes: PRCC (blue), ccRCC (red), and solid tissue normal (green). In particular, the two disease classes form two well-defined clusters for most of the patients, but some of them appear to be mixed. Some of these mixed samples are differently assigned by spectral clustering compared to ground truth labels. Looking at the heatmap, these samples seem to not belong to any of the present classes and show a big heterogeneity among themselves as well. A certain grade of heterogeneity is also shown by the normal samples, but not enough to not assign them to the same cluster. Heatmaps for the remaining datasets are provided in Additional file 8.

## Conclusions

In this paper, we describe a methodological approach for clustering of biological networks obtained by the integration of genome-scale metabolic models with gene expression data. Each sample in a dataset is described by a network, whose nodes are metabolites connected by an edge when involved in the same reaction. The edge weights are derived by the abundance of the enzymes catalyzing the associated reaction. The networks are then simplified, summarizing them into supernodes, to reduce the computational complexity of the clustering algorithm, which uses an adjacency matrix containing the distances between all pairs of networks. We show that the performance of this approach on literature data is competitive with the clustering of raw data, with the advantage of highlighting the cross-talk between different metabolic modules and pathways. Future work will be performed to extract biological information linked to the connection between the supernodes which define the differences among the classes. Furthermore, given the heterogeneity of tumors, clinical information and further subclasses annotations will be exploited in networks analysis.

## Supplementary information

**Additional file 1** List of recurrent metabolites removed from the network. The file AdditionalFile1.xlsx contains a list of all the recurrent metabolites which were removed during network construction.

**Additional file 2** Connected components size. The file AdditionalFile2.xlsx provides tables showing number of connected components and relative nodes from the four datasets networks.

**Additional file 3** Clustering Results. The file AdditionalFile3.pdf provides spectral clustering results with different numbers *k* of supernodes for network summarization.

**Additional file 4** KEGG term enrichment from the 50 clusters of the Kidney metabolic network. The file AdditionalFile4.xlsx provides the list of KEGG terms enriched from the enzymes belonging to the different clusters in the kidney metabolic network.

**Additional file 5** Clustering metrics. The file AdditionalFile5.pdf provides an in depth description of all the metrics adopted for clustering evaluation.

**Additional file 6** Matlab scripts for clustering evaluation. The file AdditionalFile6.zip provides a (zipped) set of Matlab scripts, used for clustering evaluation.

**Additional file 7** t-SNE-based visual representations. The file AdditionalFile7.pdf provides the t-SNE visual representations for expression, whole graph, and summarized graph data in Breast Microarray, Breast RNAseq, and Lung datasets.

**Additional file 8** Heatmap representations. The file AdditionalFile8.pdf provides the heatmap representations for summarized graphs in Breast Microarray, Breast RNAseq, and Lung datasets.

## Data Availability

The data used in the present study are publicly available and have been downloaded from three main public data repositories. In detail: the gene expression microarray data are available at the Gene Expression Omnibus portal (https://www.ncbi.nlm.nih.gov/gds) under the accession number GSE78958; gene expression RNA-seq data are available at the Genomic Data Commons Data Portal (https://portal.gdc.cancer.gov) under the cancer projects indicated in the “Data” paragraph of Materials and Methods; the metabolic models can be retrieved from the HMA database at http://www.metabolicatlas.org.

## Abbreviations

TCGA: The cancer genome atlas
HMA: Human metabolic atlas
GSM: Genome scale metabolic model
TCGA-BRCA: The cancer genome atlas breast invasive carcinoma
LUSC: Lung squamous cell carcinoma
LUAD: Lung adenocarcinoma
KIRC: Kidney renal clear cell carcinoma
KIRP: Kidney renal papillary cell carcinoma
ccRCC: Clear cell renal cell carcinoma
PRCC: Papillary renal cell carcinoma
SBML: Systems biology markup language
t-SNE: T-distributed stochastic neighbor embedding
RI: Rand’s index
ARI: Adjusted rand’s index
MR: Misclassification rate
F1: F-Measure
FMI: Fowlkes-Mallows index
CA: Cluster accuracy
NMI: Normalized mutual information
AMI: Adjusted mutual information
