# Correction to: Clustering analysis of tumor metabolic networks

**DOI:** 10.1186/s12859-020-03840-8

**Published:** 2020-11-02

**Authors:** Ichcha Manipur, Ilaria Granata, Lucia Maddalena, Mario R. Guarracino

**Affiliations:** 1 Institute for High-Performance Computing and Networking, National Research Council, Via P. Castellino 111, 80131 Naples, Italy; 2 HSE - National Research University Higher School of Economics, LATNA Laboratory, 13 Rodionova Ulitsa, Nizhny Novgorod, Russia

## Correction to: BMC Bioinformatics 2020, 21(Suppl 10):349 10.1186/s12859-020-03564-9

Following publication of the original article [1], the authors identified that all the figures in additional file 7 and additional file 8 were missing.

The complete and correct Additional file 7 and Additional file 8 are included in the Supplementary information section of this correction.

## Supplementary information

**Additional file 7**. t-SNE-based visual representations. The file AdditionalFile7.pdf provides the t-SNE visual representations for expression, whole graph, and summarized graph data in Breast Microarray, Breast RNAseq, and Lung datasets.

**Additional file 8**. Heatmap representations. The file AdditionalFile8.pdf provides the heatmap representations for summarized graphs in Breast Microarray, Breast RNAseq, and Lung datasets.

