# Supplementary material for: Clustering analysis of tumor metabolic networks
Source: BMC Bioinformatics. 2020 Aug 25;21(Suppl 10):349. doi: 10.1186/s12859-020-03564-9 (PMC7446216; doi:10.1186/s12859-020-03564-9)
Supplement: Supplementary file 3 — Additional file 3 Clustering Results. The file AdditionalFile3.pdf provides spectral clustering results with different numbers k of supernodes for network summarization. [file 12859_2020_3564_MOESM3_ESM.pdf]

ADDITIONAL FILE 3

Clustering analysis of tumor metabolic networks

Ichcha Manipur, Ilaria Granata, Lucia Maddalena and Mario R. Guarracino

Full list of author information is available at the end of the article

Additional File 3 — Clustering Results

Table 1 Performance of spectral clustering algorithm on the four datasets.

Clustering data	RI	ARI	MR	F_1	FMI	CA	NMI	AMI
				Breast Microarray				
Expression	47.28	-2.06	52.72	43.70	44.66	44.02	13.84	10.84
Whole graph	67.30	26.25	32.70	49.77	50.28	55.98	30.10	27.56
50	67.1	24.76	32.9	47.52	48.37	50.48	30.02	27.03
100	63.48	17.39	36.52	43.47	43.97	48.09	25.48	23.18
150	59.84	10.47	40.16	40.17	40.4	42.58	17.58	16.06
200	63.01	19.38	36.99	47.98	48.03	50.48	27.57	26.54
250	64.65	21.04	35.35	47.07	47.37	44.98	28.48	26.48
300	66.05	26.09	33.95	52.40	52.45	53.11	19.96	19.00
				Breast RNAseq				
Expression	53.05	6.21	46.95	60.99	61.90	62.59	10.08	8.14
Whole graph	52.81	5.62	47.19	52.85	52.85	62.09	4.30	4.11
50	54.44	8.88	45.56	54.46	54.46	65.09	6.74	6.56
100	51.63	3.27	48.37	52.78	52.8	59.35	2.76	2.53
150	52.13	4.25	47.87	52.03	52.03	60.6	3.27	3.1
200	49.91	-0.17	50.09	49.98	49.98	51.37	0.05	-0.13
250	52.02	4.05	47.98	52.09	52.09	60.35	3.14	2.96
300	54.90	9.81	45.10	54.82	54.82	65.84	7.37	7.20
				Lung				
Expression	89.79	79.17	10.21	88.11	88.12	94.07	78.31	77.83
Whole graph	89.56	78.73	10.44	87.91	87.92	93.77	75.94	75.02
50	67.77	33.51	32.23	60.82	60.86	72.4	40.73	38.7
100	70.73	41.19	29.27	67.83	67.97	79.53	49.67	47.55
150	82.43	64.21	17.57	79.68	79.69	89.02	63.67	62.93
200	75.6	50.35	24.4	71.88	71.89	83.38	53.99	53.52
250	86.18	71.88	13.82	84.07	84.09	91.69	72.01	71.04
300	87.56	74.64	12.44	85.56	85.57	92.58	72.72	72.00
				Kidney				
Expression	88.67	76.42	11.33	85.88	85.88	91.97	70.89	70.66
Whole graph	87.91	74.94	12.09	85.11	85.12	91.64	69.50	68.80
50	76.49	51.51	23.51	71.46	71.5	80.94	47.01	45.01
100	86.49	71.94	13.51	83.27	83.27	90.3	65.96	65.47
150	82.25	62.83	17.75	77.48	77.49	85.62	56.97	56.1
200	82.61	63.47	17.39	77.73	77.75	85.62	56.78	55.62
250	88.09	75.42	11.91	85.52	85.56	91.64	70.00	68.80

(All values have been multiplied by 100).
