# Supplementary material for: Clustering analysis of tumor metabolic networks
Source: BMC Bioinformatics. 2020 Aug 25;21(Suppl 10):349. doi: 10.1186/s12859-020-03564-9 (PMC7446216; doi:10.1186/s12859-020-03564-9)
Supplement: Supplementary file 5 — Additional file 5 Clustering metrics. The file AdditionalFile5.pdf provides an in depth description of all the metrics adopted for clustering evaluation. [file 12859_2020_3564_MOESM5_ESM.pdf]

ADDITIONAL FILE 5

Clustering analysis of tumor metabolic networks

Ichcha Manipur, Ilaria Granata, Lucia Maddalena and Mario R. Guarracino

Full list of author information is available at the end of the article

Additional File 5 — Clustering Metrics

The evaluation of clustering algorithms is generally carried out in terms of *internal* and *external* validation indices [1]. Internal indices aim at evaluating the goodness of a computed data partition using quantities and features extracted from the data. On the other side, external indices are based on the existence of a given ground truth data partition and aim at evaluating how accurately a clustering technique partitions the data as compared to the ground truth.

In our applications, we are given ground truths for all the considered datasets and, rather than evaluating a clustering technique *per se*, we are interested in evaluating its ability in partitioning the given data for the problems at hand. Therefore, we select an extended set of external accuracy metrics often adopted for clustering evaluation [1, 2, 3, 4, 5], described in detail in the following.

Notation

Most of these metrics are defined in terms of the total number of True Positives (TP), False Positives (FP), False Negatives (FN), and True Negatives (TN), formalized as follows.

Given a set of n elements $S = \{s_1, \ldots, s_n\}$, a ground truth partition of S into c clusters $GT = \{GT_1, \ldots, GT_c\}$, and a partition of S into c clusters $CP = \{CP_1, \ldots, CP_c\}$ computed with any clustering technique, we define the following:

- TP = number of pairs of elements in S that are in the same cluster in CP and in the same cluster in GT

  $$TP = |\{(s_i, s_j) : s_i, s_j \in CP_l \wedge s_i, s_j \in GT_m\}|$$

  for some $i, j \in \{1, \ldots, n\}$ and $l, m \in \{1, \ldots, c\}$, where $|\cdot|$ indicates the cardinality of a set and $\wedge$ indicates the logical AND.
- TN = number of pairs of elements in S that are in different clusters in CP and in different clusters in GT

  $$TN = |\{(s_i, s_j) : s_i \in CP_{l_1} \wedge s_j \in CP_{l_2} \wedge s_i \in GT_{m_1} \wedge s_j \in GT_{m_2}\}|$$

  for some $i, j \in \{1, \ldots, n\}$, $l_1, l_2, m_1, m_2 \in \{1, \ldots, c\}$, $l_1 \neq l_2$, and $m_1 \neq m_2$.
- FP = number of pairs of elements in S that are in the same cluster in CP but in different clusters in GT

  $$FP = |\{(s_i, s_j) : s_i, s_j \in CP_l \wedge s_i \in GT_{m_1} \wedge s_j \in GT_{m_2}\}|$$

  for some $i, j \in \{1, \ldots, n\}$, $l, m_1, m_2 \in \{1, \ldots, c\}$, and $m_1 \neq m_2$.

- FN = number of pairs of elements in S that are in different clusters in CP but in the same cluster in GT

  $$FN = |\{(s_i, s_j) : s_i \in CP_{l_1} \wedge s_j \in CP_{l_2} \wedge s_i, s_j \in GT_m\}|$$

  for some $i, j \in \{1, \ldots, n\}$, $l_1, l_2, m \in \{1, \ldots, c\}$, and $l_1 \neq l_2$.

The total number of possible pairs is given by

$$\binom{n}{2} = \frac{n(n-1)}{2}.$$

Intuitively, $TP+TN$ can be considered as the number of agreements between the computed partition CP and the ground truth partition GT, i.e., the number of correct decisions made by the clustering technique of assigning two similar elements to the same cluster (TP) or of assigning two dissimilar elements to different clusters (TN). Likewise, FP+FN can be considered as the number of disagreements between CP and GT, i.e., the number of wrong decisions of assigning two dissimilar elements to the same cluster (FP) or of assigning two similar elements to different clusters (FN).

Rand Index (RI) and Adjusted Rand Index (ARI)

Named by [6] after W.R. Rand [7], the Rand Index measures the percentage of decisions taken by the clustering technique under exam that are correct, i.e., its accuracy

$$RI = \frac{TP + TN}{TP + FP + FN + TN} = \frac{2(TP + TN)}{n(n-1)}.$$

RI assumes values in [0,1].

As observed in [3, 6], RI does not guarantee that random partitions will get a value close to zero. To counter this effect, some authors prefer to adopt the so-called Adjusted Rand Index, defined as

$$ARI = \frac{RI - E[RI]}{\max(RI) - E[RI]},$$

where $E[RI]$ indicates the expected RI of a random partition. ARI assumes values in [-1,1]; negative values indicate that the computed partition is worse than a random partition.

Misclassification Rate (MR)

The Misclassification Rate measures the percentage of wrong decisions taken by a clustering technique [4], computed as

$$MR = 1 - RI = \frac{FP + FN}{TP + FP + FN + TN}.$$

It assumes values in [0,1].

F-Measure (F_1)
The F-measure, also known as Figure of Merit, is the weighted harmonic mean of *Precision* and *Recall*

$$F_1 = \frac{2Precision \cdot Recall}{Precision + Recall},$$

where *Precision* and *Recall* are defined as

$$Precision = \frac{TP}{TP + FP} \qquad Recall = \frac{TP}{TP + FN}.$$

F_1 assumes values in [0,1].

Fowlkes-Mallows Index (FMI)
The Fowlkes-Mallows index [8] is defined as the geometric mean of *Precision* and *Recall*

$$FMI = \frac{TP}{\sqrt{(TP + FP) \cdot (TP + FN)}}.$$

It assumes values in [0,1].

Cluster Accuracy (CA)
Cluster Accuracy is defined [1] as

$$CA = \frac{1}{n} \sum_{i=1}^{c} f(CP_i|GT),$$

where $f(CP_i|GT)$ indicates the number of elements of cluster CP_i whose label corresponds to the ground truth label most frequent in the cluster. CA assumes values in (0,1].

Normalized Mutual Information (NMI) and Adjusted Mutual Information (AMI)
The Normalized Mutual Information measures the amount of statistical information shared by random variables representing the cluster assignments and the ground truth label assignments of the elements. Given the computed partition CP of the set S into c clusters, its entropy is the amount of uncertainty for that partition, defined [3] as

$$H(CP) = -\sum_{i=1}^{c} p(i) \log(p(i)),$$

where $p(i) = |CP_i|/n$ is the probability that an element picked at random from CP falls into class CP_i. Likewise, let the entropy of the ground truth partition GT be given by

$$H(GT) = -\sum_{j=1}^{c} p'(j) \log(p'(j)),$$

where $p'(j) = |GT_j|/n$ is the probability that an element picked at random from GT falls into class GT_j. The mutual information between CP and GT is computed as

$$MI = \sum_{i=1}^{c}\sum_{j=1}^{c} p(i,j) \log\left(\frac{p(i,j)}{p(i)p'(j)}\right),$$

where $p(i,j) = |CP_i \cap GT_j|/n$ is the probability that an element picked at random falls into both classes CP_i and GT_j. The Normalized Mutual Information is defined [9] as

$$NMI = \frac{MI}{\sqrt{H(CP)H(GT)}}$$

and assumes values in [0,1].

As with the Rand Index, NMI is not adjusted for chance. To counter this effect, the Adjusted Mutual Information can be adopted [10, 11, 12], defined as

$$AMI = \frac{MI - E[MI]}{\sqrt{H(CP)H(GT)} - E[MI]},$$

where $E[MI]$ indicates the expected MI of a random partition. AMI assumes values in [-1,1].

References

1. Fahad, A., Alshatri, N., Tari, Z., Alamri, A., Khalil, I., Zomaya, A.Y., Foufou, S., Bouras, A.: A survey of clustering algorithms for big data: Taxonomy and empirical analysis. IEEE Transactions on Emerging Topics in Computing **2**(3), 267–279 (2014). doi:10.1109/TETC.2014.2330519
2. Pedregosa, F., Varoquaux, G., Gramfort, A., Michel, V., Thirion, B., Grisel, O., Blondel, M., Prettenhofer, P., Weiss, R., Dubourg, V., Vanderplas, J., Passos, A., Cournapeau, D., Brucher, M., Perrot, M., Duchesnay, E.: Scikit-learn: Machine learning in Python. J. Mach. Learn. Res. **12**, 2825–2830 (2011)
3. INRIA, et al.: scikit-learn. Machine Learning in Python. http://scikit-learn.org/stable/modules/clustering.html
4. Yassouridis, C., Leisch, F.: Benchmarking different clustering algorithms on functional data. Advances in Data Analysis and Classification **11**(3), 467–492 (2017). doi:10.1007/s11634-016-0261-y
5. Manning, C.D., Raghavan, P., Schutze, H.: Introduction to Information Retrieval. Cambridge University Press (2008). http://nlp.stanford.edu/IR-book/information-retrieval-book.html
6. Hubert, L., Arabie, P.: Comparing partitions. Journal of Classification **2**(1), 193–218 (1985). doi:10.1007/BF01908075
7. Rand, W.M.: Objective criteria for the evaluation of clustering methods. Journal of the American Statistical Association **66**(336), 846–850 (1971)
8. Fowlkes, E.B., Mallows, C.L.: A method for comparing two hierarchical clusterings. Journal of the American Statistical Association **78**(383), 553–569 (1983). doi:10.1080/01621459.1983.10478008
9. Strehl, A., Ghosh, J.: Cluster ensembles — a knowledge reuse framework for combining multiple partitions. J. Mach. Learn. Res. **3**, 583–617 (2002)
10. Vinh, N.X., Epps, J.: A novel approach for automatic number of clusters detection in microarray data based on consensus clustering. In: 2009 Ninth IEEE International Conference on Bioinformatics and BioEngineering, pp. 84–91 (2009). doi:10.1109/BIBE.2009.19
11. Vinh, N.X., Epps, J., Bailey, J.: Information theoretic measures for clusterings comparison: Is a correction for chance necessary? In: Proceedings of the 26th Annual International Conference on Machine Learning. ICML '09, pp. 1073–1080. ACM, New York, NY, USA (2009). doi:10.1145/1553374.1553511. http://doi.acm.org/10.1145/1553374.1553511
12. Vinh, N.X., Epps, J., Bailey, J.: Information theoretic measures for clusterings comparison: Variants, properties, normalization and correction for chance. J. Mach. Learn. Res. **11**, 2837–2854 (2010)
13. CDSLab: Matlab Scripts for Evaluating Clustering Algorithms Using External Validation Indices. http://www.na.icar.cnr.it/~maddalena.l/ClusteringEval.html
