# Supplementary material for: Clustering analysis of tumor metabolic networks
Source: BMC Bioinformatics. 2020 Aug 25;21(Suppl 10):349. doi: 10.1186/s12859-020-03564-9 (PMC7446216; doi:10.1186/s12859-020-03564-9)
Supplement: Supplementary file 8 — Additional file 8 Heatmap representations. The file AdditionalFile8.pdf provides the heatmap representations for summarized graphs in Breast Microarray, Breast RNAseq, and Lung datasets. [file 12859_2020_3564_MOESM8_ESM.pdf]

ADDITIONAL FILE 8

Clustering analysis of tumor metabolic networks

Ichcha Manipur, Ilaria Granata, Lucia Maddalena and Mario R. Guarracino*

*Correspondence:
mario.guarracino@cnr.it
Full list of author information is available at the end of the article

Additional File 8 — Heatmap representations

Figure 1 Breast microarray dataset Heatmap representation of the clustering performed on the distance matrix of the Breast microarray summarized network.

Figure 2 Breast RNAseq dataset Heatmap representation of the clustering performed on the distance matrix of the Breast RNAseq summarized network.

Figure 3 Lung dataset Heatmap representation of the clustering performed on the distance matrix of the Lung summarized network.
