# Supplementary material for: Correction to: Clustering analysis of tumor metabolic networks
Source: BMC Bioinformatics. 2020 Nov 2;21:494. doi: 10.1186/s12859-020-03840-8 (PMC7604946; doi:10.1186/s12859-020-03840-8)
Supplement: Supplementary file 1 — Additional file 7. t-SNE-based visual representations. The file AdditionalFile7.pdf provides the t-SNE visual representations for expression, whole graph, and summarized graph data in Breast Microarray, Breast RNAseq, and Lung datasets. [file 12859_2020_3840_MOESM1_ESM.pdf]

ADDITIONAL FILE 7

Clustering analysis of tumor metabolic networks

Ichcha Manipur, Ilaria Granata, Lucia Maddalena and Mario R. Guarracino*

*Correspondence:
mario.guarracino@cnr.it
Full list of author information is available at the end of the article

Additional File 7 — t-SNE-based visual representations

T-distributed Stochastic Neighbor Embedding (t-SNE) is a nonlinear dimensionality reduction technique that allows embedding of high-dimensional data for visualization in a low-dimensional space of two or three dimensions [1]. It models each high-dimensional sample by a two- or three-dimensional point in such a way that similar samples are modeled by nearby points and dissimilar samples are modeled by distant points with high probability. It is capable of retaining the local structure of the high-dimensional data, while also revealing some important global structure, such as the presence of clusters at several scales. The algorithm is non-linear and adapts to the underlying data, iteratively performing different transformations on different regions.

Here, we provide the t-SNE-based 2D visual representations for the Breast (Fig. 1), Breast RNAseq (Fig. 2), and Lung (Fig. 3) datasets considered in our paper. They have been obtained using the Rtsne R function providing the Barnes-Hut implementation of t-SNE, using the Euclidean distance. The following parameters have been experimentally chosen:

- initial_dims = 50: Number of dimensions to be retained in the initial PCA step for initially reducing the data dimensionality;
- perplexity = 20: Number of local neighbors of each point. It balances attention between local and global aspects of the data;
- exaggeration_factor = 12: Size of natural clusters in data;
- theta = 0.5: Accuracy trade-off for the Barnes-Hut algorithm, that performs an approximate optimization of the Kullback-Leibler divergence of distributions between the original space and the embedded space;
- max_iter = 1000: Maximum number of optimization iterations;
- normalize = 1: Data are normalized internally prior to distance calculations;
- eta = 200: Learning rate for the optimization process.

References

1. van der Maaten, L., Hinton, G.: Visualizing data using t-SNE. Journal of Machine Learning Research **9**, 2579–2605 (2008)

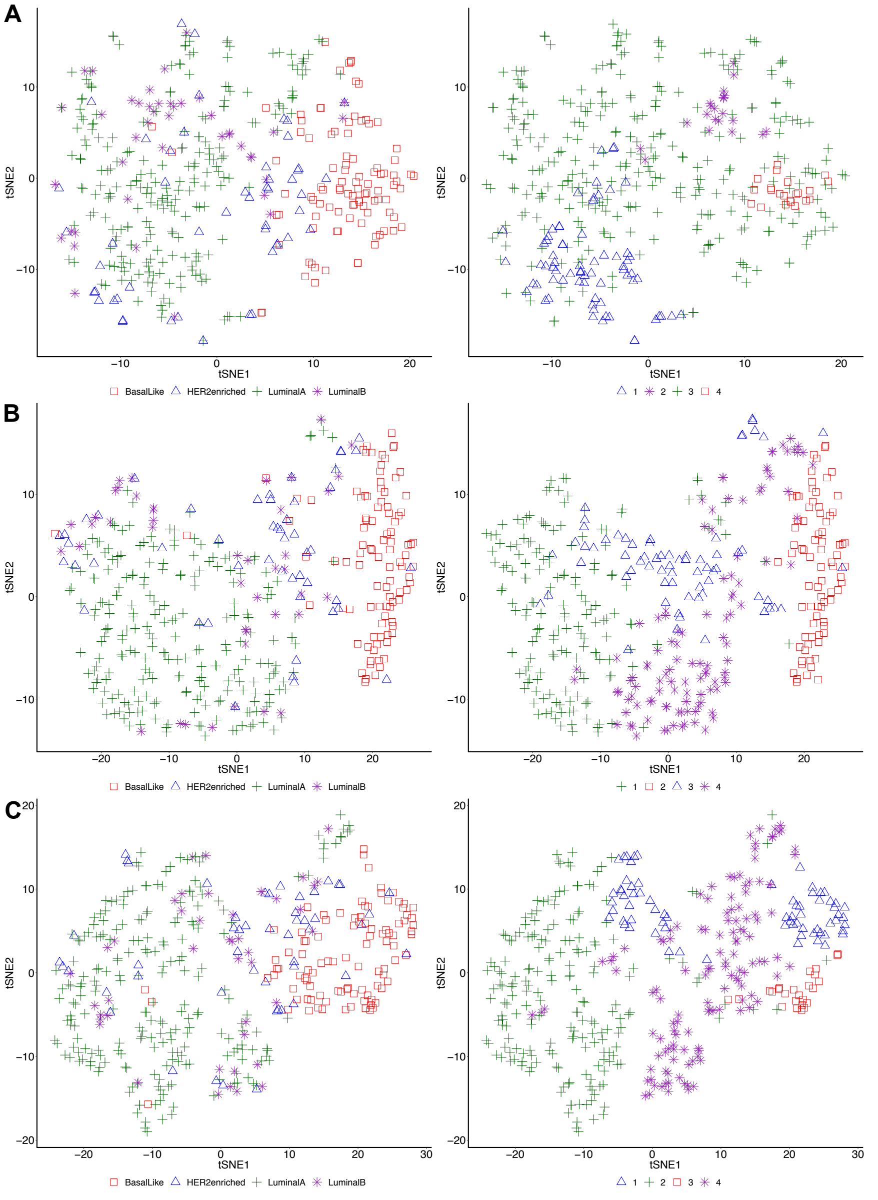

Figure 1 Breast microarray dataset t-SNE representation of gene expression data (A) and distance matrices obtained from whole (B) and compressed (C) graphs. The panel on the left shows the ground truth labels of the different classes and the right panel shows the labels assigned by spectral clustering.

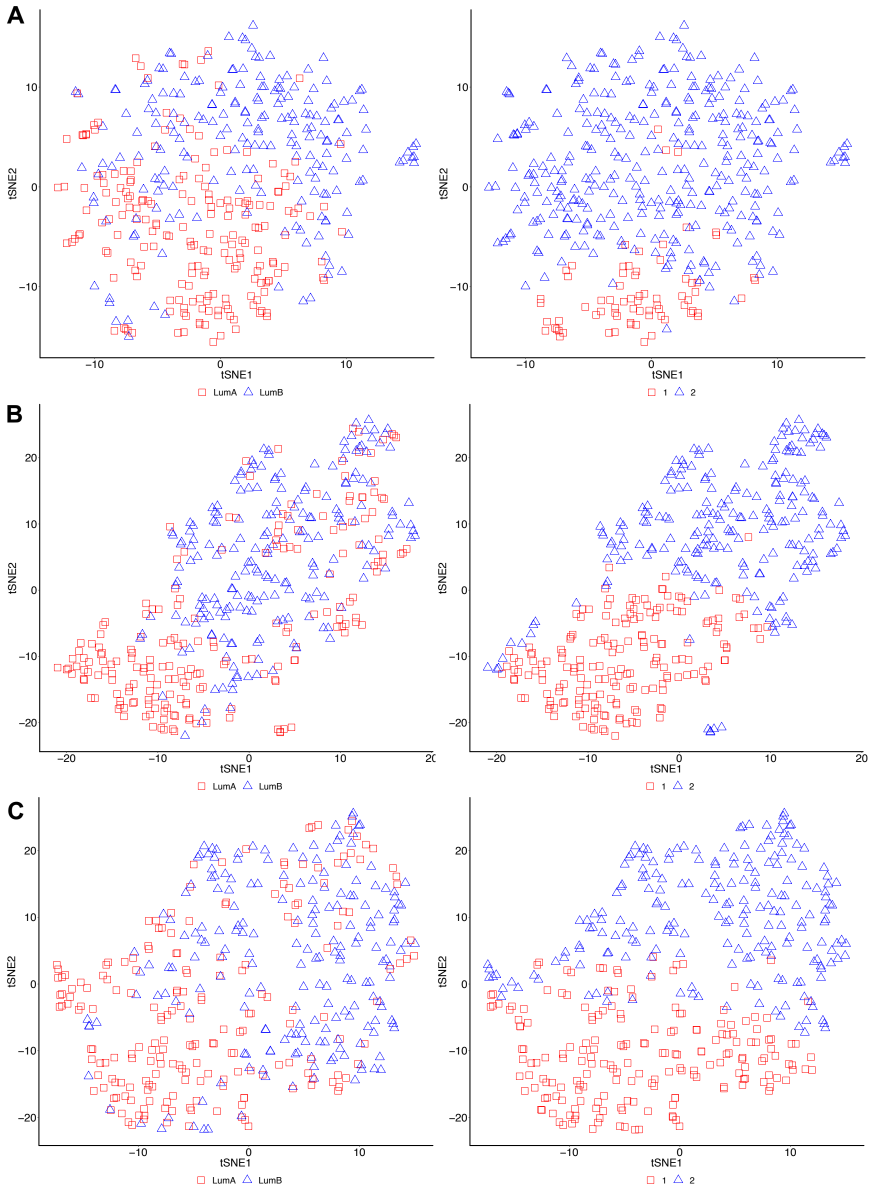

Figure 2 Breast RNAseq dataset t-SNE representation of gene expression data (A) and distance matrices obtained from whole (B) and compressed (C) graphs. The panel on the left shows the ground truth labels of the different classes and the right panel shows the labels assigned by spectral clustering.

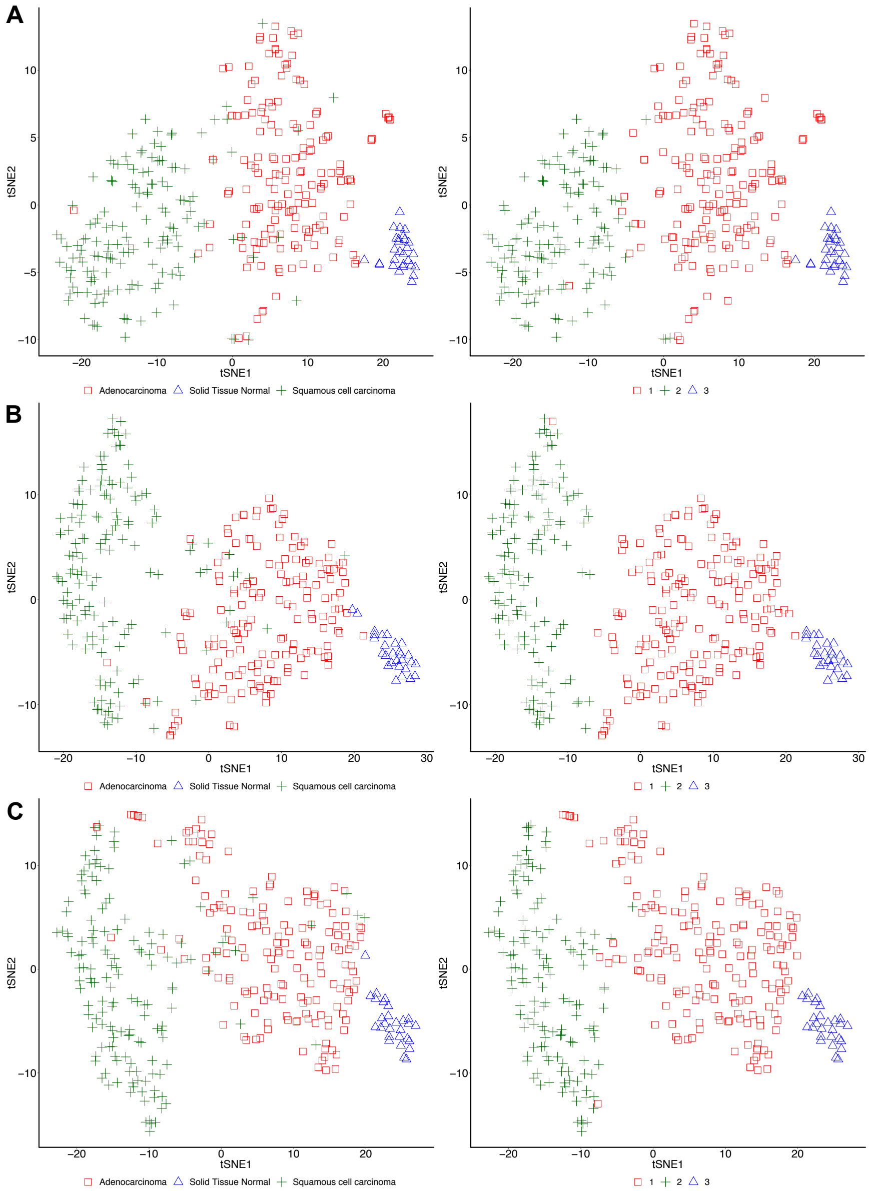

Figure 3 Lung dataset t-SNE representation of gene expression data (A) and distance matrices obtained from whole (B) and compressed (C) graphs. The panel on the left shows the ground truth labels of the different classes and the right panel shows the labels assigned by spectral clustering.
