# Supplementary material for: Correction to: Clustering analysis of tumor metabolic networks
Source: BMC Bioinformatics. 2020 Nov 2;21:494. doi: 10.1186/s12859-020-03840-8 (PMC7604946; doi:10.1186/s12859-020-03840-8)
Supplement: Supplementary file 2 — Additional file 8. Heatmap representations. The file AdditionalFile8.pdf provides the heatmap representations for summarized graphs in Breast Microarray, Breast RNAseq, and Lung datasets. [file 12859_2020_3840_MOESM2_ESM.pdf]

ADDITIONAL FILE 8

Clustering analysis of tumor metabolic networks

Ichcha Manipur, Ilaria Granata, Lucia Maddalena and Mario R. Guarracino*

*Correspondence:
mario.guarracino@cnr.it
Full list of author information is available at the end of the article

Additional File 8 — Heatmap representations

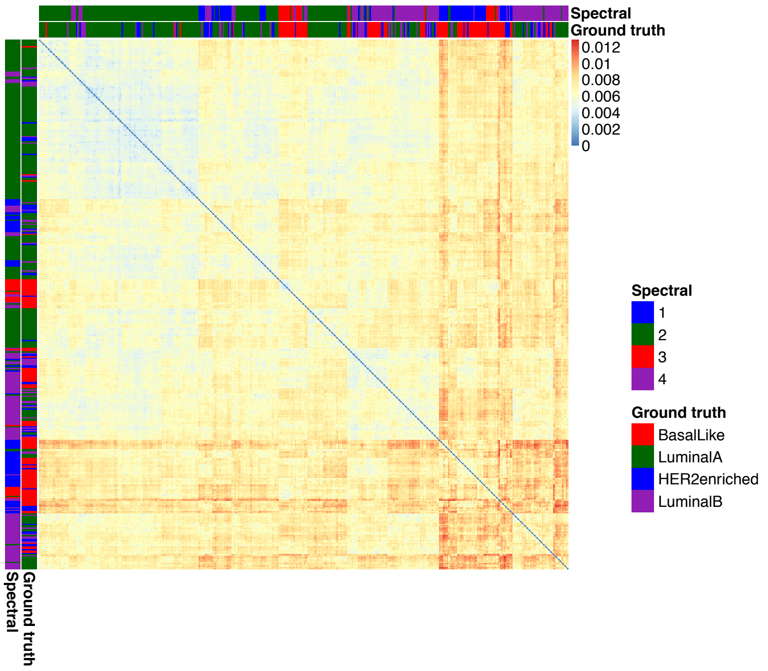

Figure 1 Breast microarray dataset Heatmap representation of the clustering performed on the distance matrix of the Breast microarray summarized network.

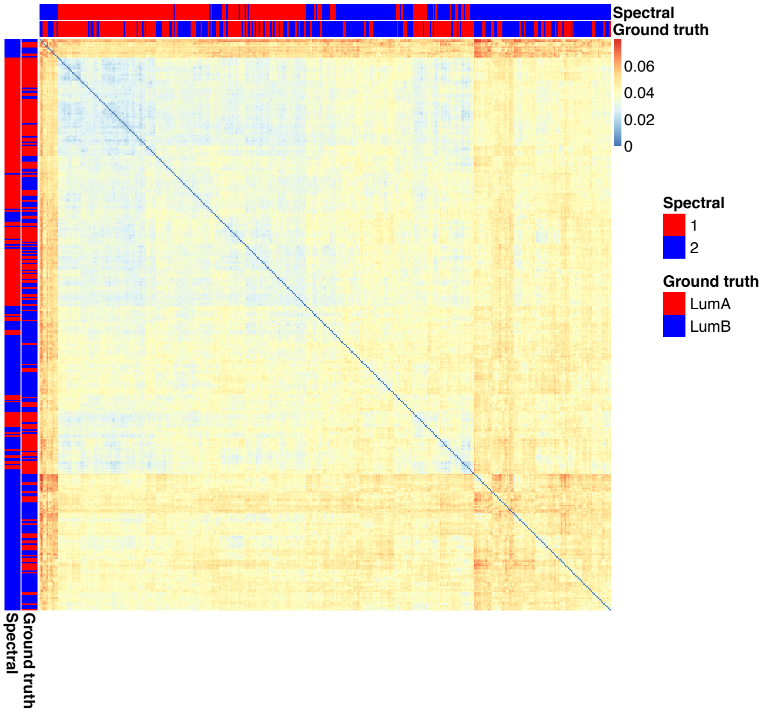

Figure 2 **Breast RNAseq dataset** Heatmap representation of the clustering performed on the distance matrix of the Breast RNAseq summarized network.

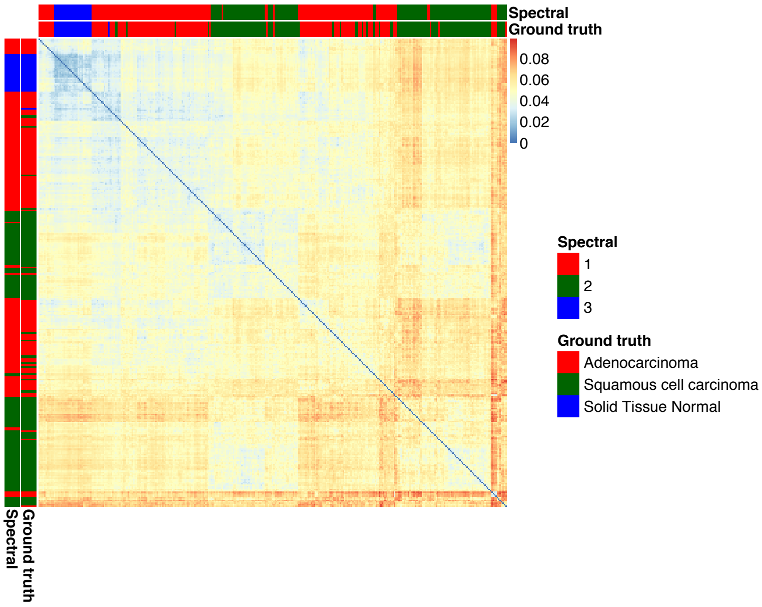

Figure 3 **Lung dataset** Heatmap representation of the clustering performed on the distance matrix of the Lung summarized network.
